# Detection of Phenylarsine Oxide in Drinking Water Using an Impedimetric Electrochemical Sensor with Gelatin-Based Solid Electrolyte Enriched with Mercaptoethanol: A Novel Prospective Green Biosensor Methodology

**DOI:** 10.1021/acsomega.2c05516

**Published:** 2022-11-15

**Authors:** Kübra Keser, Mehmet Çağrı Soylu

**Affiliations:** † Biomedical Device Technologies, Simav Vocational School, Kutahya Dumlupinar University, Simav, Kütahya43500, Turkey; ‡ Biological and Medical Diagnostic Sensors Laboratory (BioMeD Sensors Lab), Department of Biomedical Engineering, Erciyes University, Kayseri38039, Turkey

## Abstract

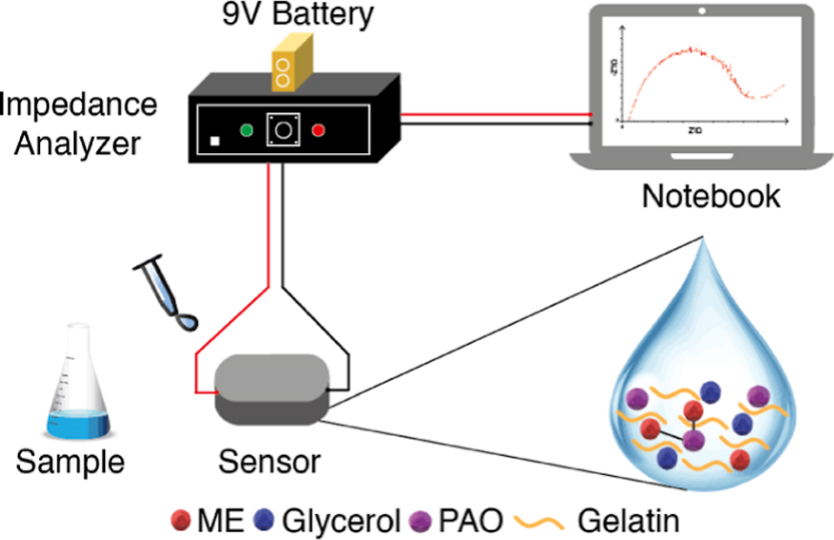

A simple, inexpensive, rapid, and label-free detection of phenylarsine oxide (PAO) in the field is a significant and unmet need because of its fatally acute and chronic effects on human health. A simple, fast, sensitive, and relatively low-cost arsenic detection system with an eco-friendly sensor could fill this gap. To monitor arsenic in situ, a reliable, portable impedimetric electrochemical sensor is the most suitable platform, which is real-time, fast, low-cost, and easy to design and use and has high sensitivity at low detection limits in the nanogram per mL range. The detection system in this study has a patent-applied green sensor with minimum harm to nature and the potential to dissolve in nature. The electrode containing 15 mL of distilled water (DIW) + 2 g gelatin + 1.75 g glycerol was determined to be the most suitable for determining the amount of inorganic arsenic in the range of 1–100 ng/mL using a gelatin-based solid electrochemical sensor enriched with 2-mercaptoethanol. Impedance measurements were performed to analyze the stability of the sensor in both deionized water and drinking water, as well as for arsenic detection. Among the procedures examined, the procedure prepared with 15 mL DIW + 2 g glycerol + 1.75 g gelatin resulted in the best stability in aqueous medium and in sensitivity with resistance changes (−Δ*R*_ct_ (%)) of 12% (±0.62%), 26% (±2.3%), and 40% (±3.8%) for the concentrations of 1, 10, and 100 ng/mL PAO in drinking water, respectively. With this detection methodology, there is the potential to detect not only arsenic but also other heavy metals in waters and different biomarkers in human fluids.

## Introduction

As one of the most abundant elements in nature, arsenic is commonly found in natural waters, soil, rocks, atmosphere, and organisms.^1^ It ranks 20th in abundance in the Earth’s crust, 14th in the seawater, and 12th in the human body.^1^ In natural waters, As exists as arsenite [As(III)], arsenate [As(V)], monomethylarsonic acid, dimethylarsinic acid, and as various organoarsenicals.^2^ Today in industrial processes of minerals, mines, volcanic movements, forest fires, pesticides, and erosion of rocks, various metalloids mix with drinking water. Its concentration in water sources has lately increased due to increasing industrial activity related to production of pigments, insecticides, herbicides, and some other related materials.^3^ Arsenic has caused many environmental concerns in personal and public health worldwide as a toxic and carcinogenic metalloid with a wide distribution in the environment.^4^ It is ingested into the human body by breathing or through skin absorption. Its toxic effect can occur in the form of acute toxicity as well as in the form of chronic toxicity. Chronic toxicity occurs with long-term exposure to low doses of arsenic from sources such as drinking water. The contamination of arsenic in water is a significant concern to human health^5−7^ as exposure can lead to a range of acute and chronic diseases, such as dysphasia, facial edema, dehydration, jaundice, and cancer.^8−14^ Arsenic compound contamination is directly associated with cancer in lungs, skin, and bladder and kidney diseases.^15^ Due to the effect of accumulation, even exposure to low levels of arsenic for a long term can lead to a variety of adverse health effects, including dermal changes and respiratory, cardiovascular, gastrointestinal, genotoxic, mutagenic, and carcinogenic effects.^1^

Arsenic, which is one of the most hazardous pollutants disrupting the natural balance, should be monitored for human and animal health because it can be found in free form in nature and because of its various toxic effects. Therefore, certain limits have been specified for arsenic levels in potable waters. In 2003, the World Health Organization (WHO) documented the inorganic arsenic in drinking water as a cancer-causing agent and announced the highest acceptable level as 10 ng/mL.^16^

In order to eliminate the danger of chronic toxicity, especially in drinking water, which is the most important source of exposure, regular, fast, low-cost detection of arsenic is important. Field test kits^17^ and atomic absorption spectrometry (AAS-HG/AAS-GF)^18^ are the most preferred laboratory-based methods for the qualitative and quantitative analyses of arsenic with a detection range of 5–500 and 0.06–0.15 ng/mL, respectively. These methods are reliable methods for arsenic detection and quantification; however, these techniques require sophisticated preparation and the usage of additional chemical agents in order to increase the sensitivity and prevent the interference.

Currently, there are various commercial methods used in the qualitative and quantitative analyses of arsenic, such as neutron activation analysis with a detection range of 0.05–0.5 ng/mL,^19^ high-pressure liquid chromatography with a detection range of 1–100 ng/mL,^20^ anodic stripping voltammetry with a detection range of 0.05–0.5 ng/mL,^21^ inductively coupled plasma-mass spectrometry with a detection range of 0.002–0.06 ng/mL.^22^ However, these laboratory-based measurements require expensive, heavy, and sophisticated instruments, high operating costs, and processes that involve complex sample preparation and cleaning steps all of which are time-consuming, unsuitable for on-site analysis, and limit the application for routine field monitoring.^2^

Electrochemical methods,^23,24^ optical methods,^25^ surface plasmon resonance (SPR) sensors,^26,27^ and nanoparticle-based sensors,^28,29^ which are under development, are some readily available techniques with high precision compared to the commercial methods, and their sensitivity limits are 0.9, 10, 10, and 5 ng/mL, respectively. In a study in which arsenic detection in aqueous solutions was performed, a composite layer of polypyrrole chitosan/cobalt ferrite nanoparticles was prepared using an electrodeposition method on a gold-coated glass slide, and it was stated that arsenic was detected with a detection limit of 1 ng/mL by creating an SPR sensor.^26^ In another study, trace amounts of arsenic(III) were detected in glassy media with a carbon electrode modified with cobalt oxide nanoparticles with a detection limit of 11 ng/mL. In terms of sensitivity, these methods are preferable.^28^ However, the disadvantages of these techniques are advanced groundwork, such as adequate electrical or optical insulation and appropriate surface functionalization.^13,25,28^

Electrochemical analysis methods are used for the determination of arsenic because of the advantage of less expensive instrumentation and being suitable for the possibility of applying portable analytical units for on-site control and decentralized monitoring. Considering the recent studies that include these methods, it is seen that in one of these studies, arsenic detection was carried out in mineral water, spring water, and tap water samples in the laboratory with nano Au-CRV film-modified glassy carbon electrode (GCE). Again, in this study, it is stated that only As(III) detection signals are obtained under the predicted conditions by overcoming the interference effects with a detection limit of 0.2 ng/mL in the 4–40 ng/mL operating range.^15^ In another study, using 0.1 M HNO_3_ electrolyte and silver nanoparticles, arsenic was detected with a detection limit of 0.179 ng/mL in the operating range of 0.6–2.6 ng/mL.^30^ In the study of arsenic(III) determination on the Au electrode modified with mercaptoethylamine in a neutral environment, the working range is 0.2–300 ng/mL, while the detection limit is 0.02 ng/mL.^31^ In the study of determination of arsenic in natural waters in the study using carbon fiber ultra-microelectrodes modified with gold nanoparticles, arsenic was determined in the working range of 5–60 ng/mL with a detection limit of 0.9 ng/mL.^24^ In another study using electroactive nanocomposite electrodes in the operating range of 0.08–15 ng/mL, arsenite and arsenate were determined with a detection limit of 0.021 and 0.034 ng/mL, respectively.^32^

Electrochemical methods have high sensitivity and selectivity, can be portable, are practical and simple, and produce results in a relatively short time. Methods under development with an advanced, easy, and fast surface modification protocol will have the potential to replace commercial experiments currently available.

The development of green sensing platforms is known to be one of the most active areas of research, minimizing the use of toxic/hazardous reagents and solvent systems and further reducing the generation of chemical waste in sensor manufacturing.^33^ Biodegradable green sensors are preferred for being environmentally friendly and biocompatible with the nature and creatures. They have high precision, do not create waste, are of low cost, and are superior over traditional analytical methodologies for environmental monitoring, thereby ensuring sustainable development of electronic technologies. They are also used in applications of humidity and pressure measurements, such as cardiovascular examination, orthopedic applications, and measurement of pressure and blood flow.^34−36^

For the detection of arsenic in drinking water, the most suitable electrochemical sensor structure and electrode production method need to be determined. Impedimetric electrochemical sensor, which is one of the electrochemical techniques whose sensitivity and selectivity has been proven for the analysis of arsenic and other heavy metal ions in the last 10 years, has been preferred.

As explained in detail previously, arsenic has adverse effects. It is not possible to detect arsenic with the desired sensitivity with the current methods. Real-time, rapid, and early detection are difficult in a systematic analysis. It requires expensive and complex instrumentation with highly skilled personnel, has long detection times, and is not suitable for field measurement. Extra substance needs to be used to increase sensitivity and reduce interference. It is not portable, and it is affected by interference.

To close this already existing gap, we have developed an impedimetric electrochemical sensor and measurement system which has high sensitivity and selectivity and allows portable transport. It is simple in design, easy to use, and of low cost and has high miniaturization capabilities and speeds, with a biodegradable bioactive layer. It is real-time, fast, and capable of early detection at low detection (LD) limits.

This study focuses on the determination of the amount of inorganic arsenic in the range of 1–100 ng/mL using a gelatin-based solid electrochemical sensor enriched with 2-mercaptoethanol (ME) and the production of the most suitable electrode for this purpose. The concentration of arsenic in distilled water (DIW) and bottled water samples is 1 ng/mL and has been proven to be detectable.

## Results and Discussion

**Figure 1:**
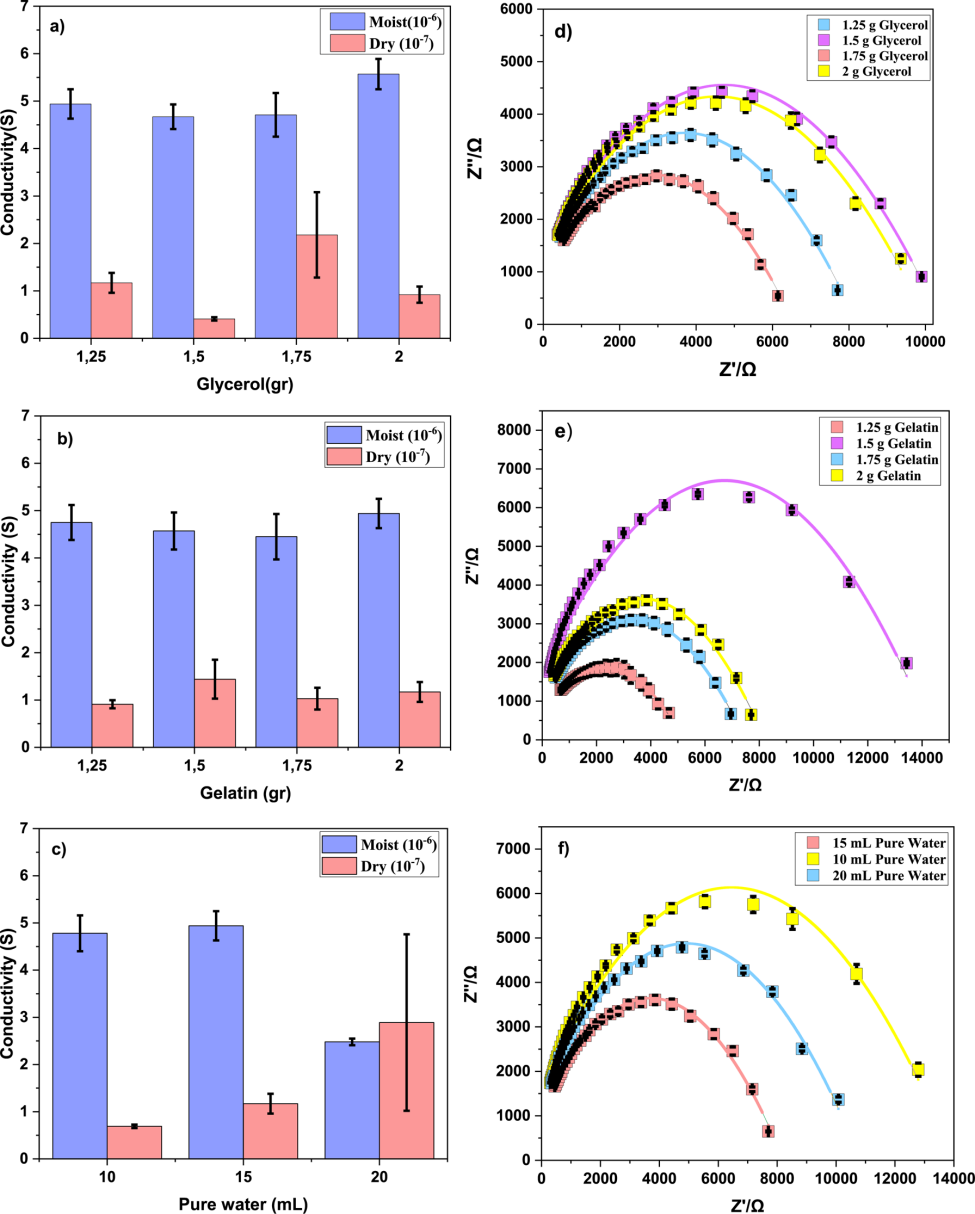
(a) Conductivity graph according to the change in the amount of glycerol (gelatin: 2 g, pure water: 15 mL). (b) Conductivity graph according to the change in the gelatin amount (glycerol: 1.25 g, pure water: 15 mL). (c) Conductivity graph according to the change in the pure water amount (gelatin: 2 g, glycerol: 1.25 g). (d) Nyquist plot depending on the change in the glycerol amount (gelatin: 2 g; pure water: 15 mL). (e) Nyquist plot depending on the change in the gelatin amount (glycerol: 1.25 g; pure water: 15 mL). (f) Nyquist plot depending on the change in the water amount (gelatin: 2 g; glycerol: 1.25 g). *Bold lines indicate fitted data.

As a result of the functionalization, which is explained in detail in the Experimental section, the desired thiol formation for the capture of phenylarsine oxide (PAO) was achieved. Conductivity measurements of the prepared moist and dry samples were taken three times, and concentration-dependent conductivity graphs were drawn using the data obtained (Figure 1). The graphs are evaluated in terms of conductivity change due to the change of glycerol, gelatin, and DIW amounts. In the case of moist and dry samples, high conductivity values are observed in moist samples. Impedance in dry samples does not change much compared to moist samples. 15 mL of pure water, 2 g of glycerol, and 1.75 g of gelatin were used in the preparation of sensors due to high conductivity values and low standard deviation. It was decided to use dry samples since there is already saturation in moist samples due to the absorption of water by the gel structure.

The procedure followed in the conductivity measurement for the samples containing different amounts of gelatin, glycerol, and DIW was also followed for the examination of the impedance change. Nyquist graphs were drawn using the results obtained (Figure 1). Looking at the Nyquist graphs in Figure 1, the *R*_ct_ values for the varying amounts of glycerol (1.25, 1.5, 1.75, and 2 g) are 7.71 kΩ (±0.011 kΩ), 9.4 kΩ (±0.025 kΩ), 6.19 kΩ (±0.029 kΩ), and 9.43 kΩ (±0.069 kΩ); the values are 4.72 kΩ (±0.111 kΩ), 13.47 kΩ (±0.045 kΩ), 0.01 kΩ (±0.031 kΩ), and 7.71 kΩ for varying amounts of gelatin (1.25, 1.5, 1.75, and 2 g); for the varying amounts of pure water (10, 15, and 20 mL), the values are 12.87 kΩ (±0.138 kΩ), 7.71 kΩ (±0.011 kΩ), and 10.10 kΩ (±0.054 kΩ). *R*_ct_ is defined as the resistance of the electrode surface to electrons.^37^ As stated in Figure 7, at high frequencies, where diffusion time constant is much longer than the signal period, the plot is described by a semicircle with the diameter given by the charge-transfer resistance *R*_ct_.^38^ Considering these results, when the general evaluation was made, as the amount of substance increased, the *R*_ct_ value increased; accordingly, the conductivity decreased, while the impedance value increased. However, at some concentration values, as stated in the literature, the saturation of the surface caused a current change and caused a decrease in the *R*_ct_ values.^39^ For this reason, a continuous increase or decrease proportional to the concentration could not be observed in the conductivity and impedance graphs.

**Figure 2:**
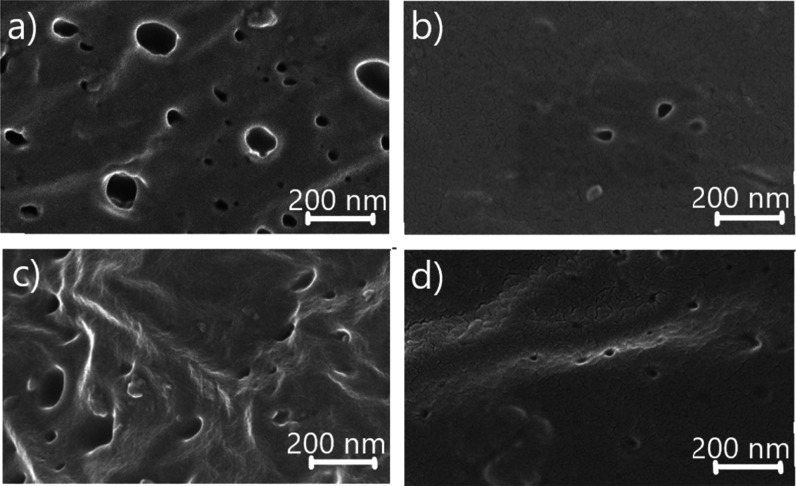
SEM images for samples. (a) 15 mL pure water + 2 g gelatin + 1.75 g glycerol. (b) 15 mL pure water + 1.75 g gelatin + 1.25 g glycerol. (c) 15 mL pure water + 2 g gelatin + 1.25 g glycerol. (d) 15 mL pure water + 1.25 g gelatin + 1.25 g glycerol.

The chemical and morphological properties of the solid electrolyte surface are directly related to the quality and sensitivity of detection. For this reason, when the graphs containing both the conductivity values and impedance change values are examined, the desired properties are seen in terms of these values, which are thought to be necessary for deciding the concentration values: 2 g gelatin + 1.75 g glycerol + 15 mL pure water, 1.25 g gelatin + 1.25 g glycerol + 15 mL pure water, 1.75 g gelatin + 1.25 g glycerol + 15 mL pure water, and 2 g gelatin + 1.25 g glycerol + 15 mL pure water. The surface morphology of dry samples was characterized by scanning electron microscopy (SEM) in order to visualize the porous structures (Figure 2). When these SEM images are examined, it is seen that the porous microstructure that allows the interaction with PAO molecules to increase in the sample structure containing 15 mL DIW + 2 g gelatin + 1.75 g glycerol is more formed and has a smoother surface morphology. The amount of gelatin, glycerol, and pure water was determined for the electrode structure to be prepared with this characterization process.

**Figure 3:**
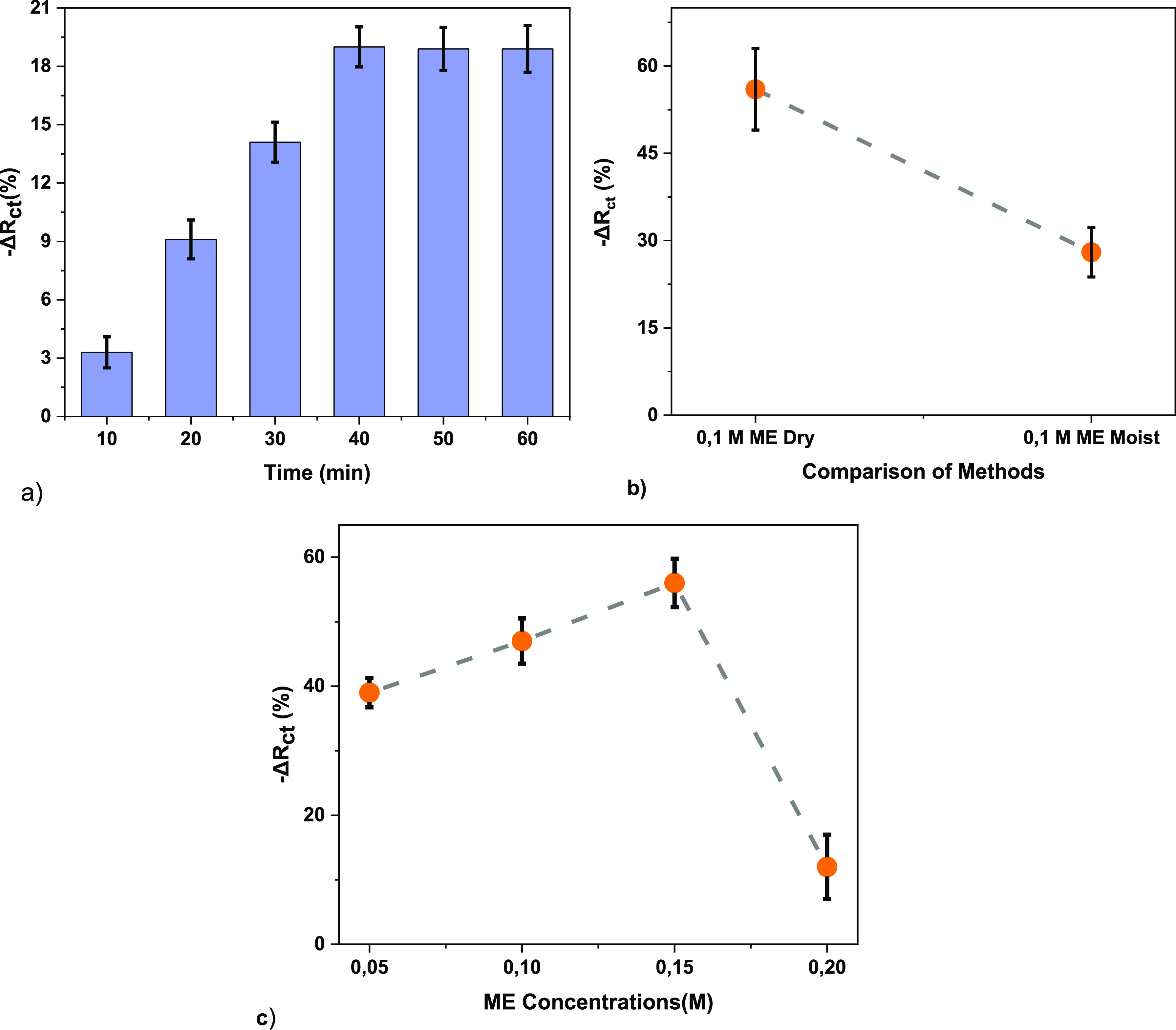
(a) Determination of detection time (Nyquist plot in 10–60 min time interval). (b) Comparative −Δ*R*_ct_ (%) plot of 0.1 M moist ME electrode and 0.1 M dry ME electrode at 100 ng/mL PAO concentration. (c) Concentration-dependent −Δ*R*_ct_ (%) graph when 100 ng/mL PAO is added to dry gelatin electrodes containing different concentrations (0.05, 0.1, 0.15, and 0.2) of ME (M).

In the determination of the detection time, the concentration of 0.1 of ME (v/v), which we demonstrated in the detection of PAO in our previous study, was studied.^38^ Nyquist graph was drawn by taking measurements from dry electrodes containing 0.1 of ME (v/v). When this graph was examined, it was concluded that 40 min was the ideal time for detection. There was no impedance change after this period since the thiol groups formed for the detection of PAO reached saturation on the surface at approximately 40 min (Figure 3a).

In the next step, in order to reveal the effect of ME electrode on detection in dry and moist samples, moist ME electrode + pure water and moist ME electrode + 100 ng/mL PAO and dry ME electrode + pure water and dry ME electrode + 100 ng/mL were studied. PAO impedance changes were examined, and the percentile *R*_ct_ change graph was drawn by using these values obtained for this experimental procedure in which both methods were compared (Figure 3b). For experiments with three replicates, when pure water was added to the moist ME electrode, the *R*_ct_ values for each replicate were 2.61 kΩ (±0.002 kΩ), 2.42 kΩ (±0.002 kΩ), and 2.53 kΩ (±0.002 kΩ); the *R*_ct_ values for the moist ME electrode + 100 ng/mL PAO were 2.15 kΩ (±0.001 kΩ), 2.73 kΩ (±0.001 kΩ), and 2.20 kΩ (±0.001 kΩ). For experiments with three replicates, when pure water was added to the dry ME electrode, the *R*_ct_ values for each replicate were 15.8 kΩ (±0.097 kΩ), 14.278 kΩ (±0.03 kΩ), and 14.54 kΩ (±0.004 kΩ). the *R*_ct_ values for the dry ME electrode + 100 ng/mL PAO are 9.91 kΩ (±0.412 kΩ), 9.31 kΩ (±0.031 kΩ), and 9.88 kΩ (±0.012 kΩ). When these results are evaluated, it can be seen that reproducibility and stability are observed for each step of the surface modification, and high conductivity values are observed in moist samples. When 100 ng/mL of PAO is added to the ME electrode surface, the conductivity increases due to the increase in current on the surface. Impedance is reduced, and it is concluded that the impedance change is higher in dry samples.

After determining the detection time, the ME concentration where the impedance change is the greatest when PAO is added to the ME electrode was determined; 100 ng/mL PAO was determined by dry gelatin electrodes containing different concentrations (0.05, 0.1, 0.15, and 0.2) of ME (M). When the graph was drawn using the results obtained, as the ME concentration increases, the *R*_ct_ values appears to be 8.38 kΩ (±0.034 kΩ), 8.70 kΩ (±0.029 kΩ), 17.66 kΩ (±0.093 kΩ), and 10.5 kΩ (±0.104 kΩ). Considering these results, it was observed that as the ME concentration increased, the conductivity increased considerably and the impedance decreased in PAO detection. This revealed that the impedance change at different concentrations of ME was directly related to the increase of S–As–S complexes on the sensor surface. However, after 0.15 of ME (v/v) concentration limit, this situation started to reverse, that is, the *R*_ct_ value increased in the Nyquist plot. The reason for this situation is the formation of clusters with increasing concentration and the corresponding decrease in conductivity.^15^ Based on these *R*_ct_ values obtained for each concentration, a concentration-dependent percentile *R*_ct_ change graph was drawn (Figure 3c).

**Figure 4:**
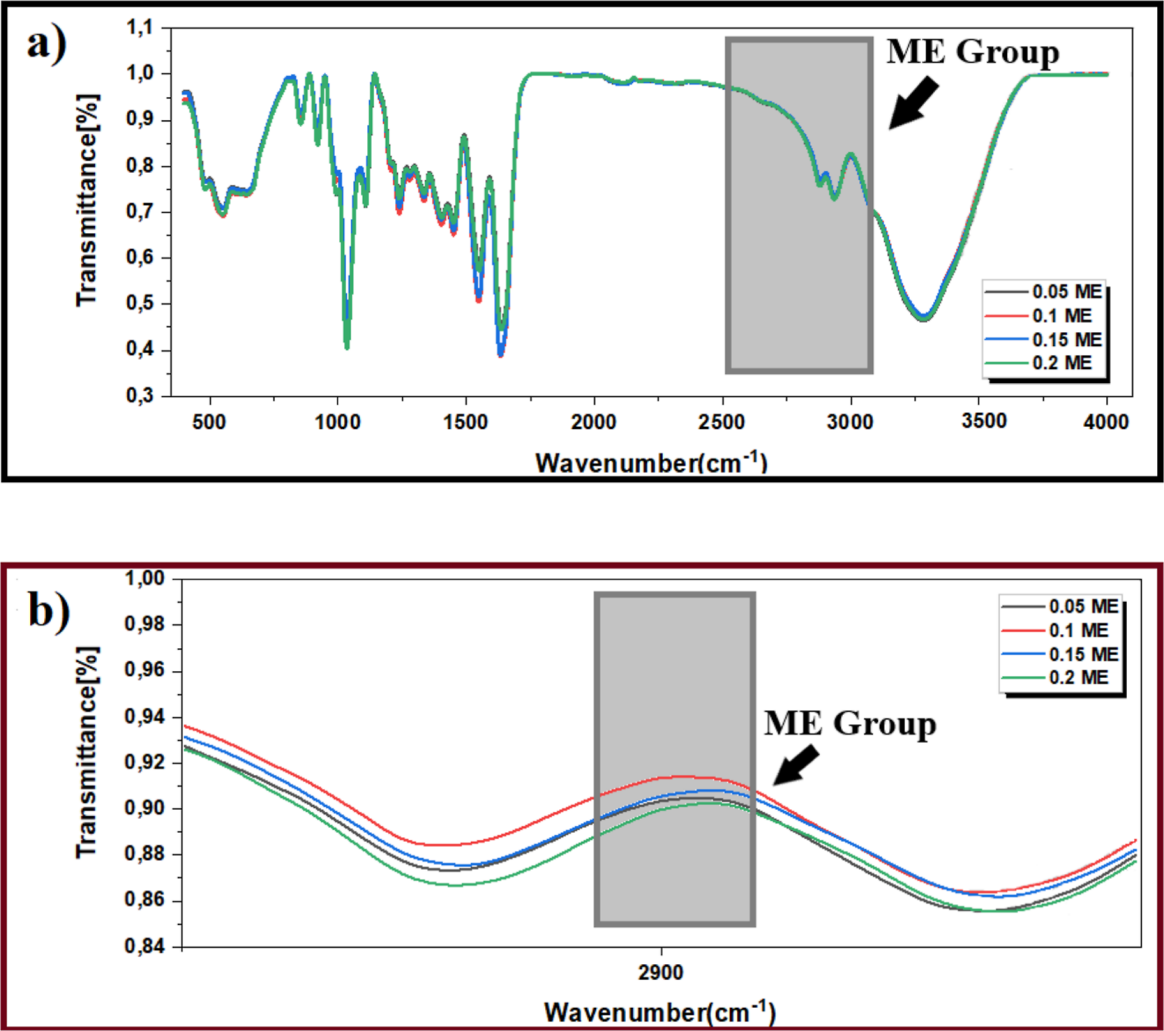
FT-IR analysis of gelatin electrodes containing different concentrations (0.05, 0.1, 0.15, and 0.2) of ME (M). (a) 400–4000 cm^–1^ wavelength range and (b) 2400–3000 cm^–1^ wavelength range, which is the specific wavelength range in which the thiol group is analyzed.

In order to observe the change of arsenic reactive SH groups in samples prepared at different ME concentrations, Fourier transform infrared spectroscopy (FT-IR) analysis was performed both in the wavelength range of 400–4000 cm^–1^ and in the wavelength range of 2400–3000 cm^–1^, which is the specific wavelength range in which the thiol group is analyzed.^40^ The peak value in the wavelength range of about 2650 cm^–1^ where the thiol group is located increases inversely with the amount of ME. When the data obtained are examined, it is seen that there are small changes between these peaks due to the interaction of the gelatin + glycerol structure with ME at different concentrations, but when we look at the transmittance value in the wavelength range of 2400–3000 cm^–1^ in general, it is seen that thiol groups are more in the structure containing 0.15 of ME (v/v) (Figure 4). Therefore, the concentration to be used in the study was determined as 0.15 M of ME.

**Figure 5:**
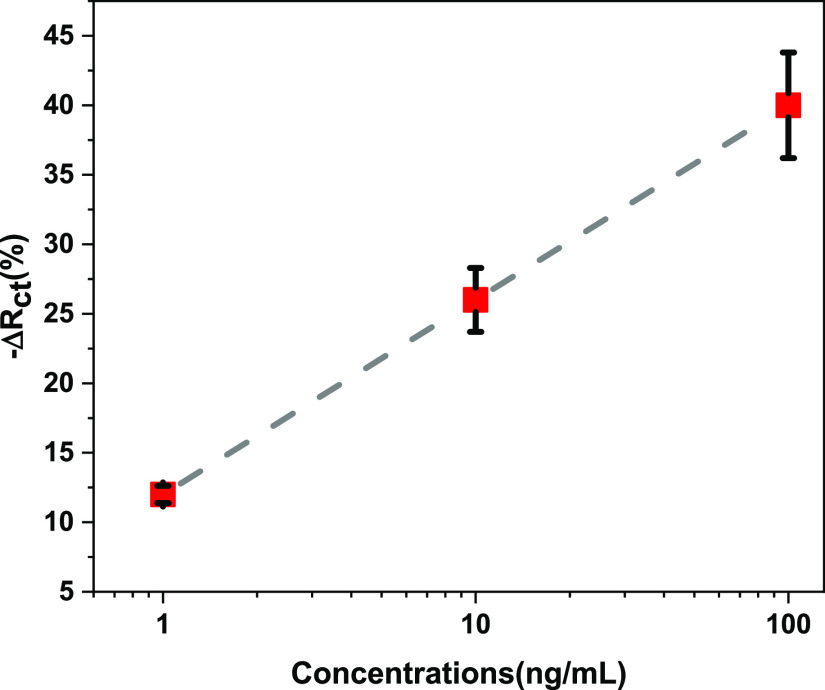
Concentration-dependent −Δ*R*_ct_ (%) graph when different concentrations (1, 10, and 100 ng/mL) of PAO were added to dry gelatin electrodes containing 0.15 M of ME.

Electrodes were prepared according to the 15 mL pure water + 2 g gelatin + 1.75 g glycerol + 45 μL ME procedure and left to dry. With these dried electrodes, measurements were taken in three repetitions for the determination of PAO at concentrations of 1–10–100 ng/mL, and the results were examined. When the Nyquist graph created with the results obtained is examined, the *R*_ct_ values when pure water is added to the created electrode are 8.98 kΩ (±0.015 kΩ), 9.58 kΩ (±0.059 kΩ), and 9.10 kΩ (±0.053 kΩ); the *R*_ct_ values for increasing PAO concentration are 9.02 kΩ (±0.049 kΩ), 7.53 kΩ (±0.041 kΩ), and 5.95 kΩ (±0.024 kΩ). Decreasing *R*_ct_ values with increasing PAO concentration are consistent with the state of the art. According to the literature, when electrochemical impedance spectroscopy (EIS) was used for detection of arsenic, the value of electron-transfer resistance (*R*_ct_) decreased with increasing As concentration. The reason for this was the absorption of As on the film surface, which further inhibited the electron-transfer kinetics on the film surface. In the same study, it was stated that the current increases as the As concentration increases.^15^ Again, in many studies in the literature, it is seen that as the As concentration increases, there is increase in current which is expressed with graphs.^23,24,31,41^ The most critical point of the detection mechanism in this study is the reaction between PAO and adjacent thiol groups. When PAO binds with thiols, two important consequences arise that allow electrochemical detection. The first is that when two closely free thiol groups capture PAO, the gelatin, which forms the main part of the solid electrode structure, is folded and subjected to mechanical stress. When the gelatin with ion-rich regions is folded, these regions get closer to each other, and their conductivity increases.^42^ Besides, the interfacial electron transfer becomes easier since this folding changes the dielectric property of the structure and the gelatin configuration.^41^ The second important effect is that water is released as a result of the reaction between PAO and thiols, which lowers the pH of the electrolyte. This situation facilitates the folding of the gelatin structure as the displacement of negative charges and positive charges will cause the electrostatic repulsion force to act.^43^ When this information is evaluated, it is seen that current will increase, impedance will decrease, and conductivity will increase depending on the increase in the concentration at a constant voltage. The change in resistance for PAO concentrations of 1, 10, and 100 ng/mL in DIW is 12% (±0.62%), 26% (±2.3%), and 40% (±3.8%) of −Δ*R*_ct_ (%), respectively, and the results are consistent with similar studies. At the same time, it is seen that standardization has been achieved in sensor production. The changing PAO concentrations in water can be determined by measuring *R*_ct_. The absorption and electrode resistance decrease with increasing PAO concentration on the electrode surface, and the current increases due to the increase in the concentration, while the impedance decreases (Figure 5).

When considering the recent state-of-the-art development of arsenic detection methods, this work is able to compete with the peer techniques in terms of both limit of detection (LOD) and linear range. However, the summarized methods require complex, troublesome, and time-consuming chemical surface modification procedures (Table 1). In response to those deficits, we were able to detect PAO with an LOD of 1 ng/mL in the linear range from 1 to 100 ng/mL in as little as 40 min by using a simple procedure with relatively inexpensive, readily available, biodegradable chemicals. In this study, in which a gelatin-based solid electrode sensor system is used for the first time, the sensor fabrication is fast and low-cost, and the detection is sensitive, fast, simple, and easy. These two factors constitute the most important points of this study. On the other hand, there are doubts about the specific operation of our sensor in real drinking water samples, where other heavy metals may interfere with the measurement. The most common heavy metals in drinking water around the world are arsenic, lead, mercury, copper, chromium, and cadmium.^44^ The interference problem of other heavy metals can be overcome by preliminary chelating treatment of the water sample using ethylenediaminetetraacetic acid, which can bind to other heavy metals except arsenic with high affinity.^45^ In addition, the arsenic specificity of the sensor can be increased by using dimercaprol, which is used in the treatment of arsenic poisoning, as a receptor.^46^

**Table 1:** Comparison of the Modification Time and Analytical Performance of Some Arsenic Detection Methods

detection mechanism	modification time	detection time	linear range (ng/mL)	LOD (ng/mL)	references
colorimetric detection and membrane removal of arsenate by a multifunctional l-arginine modified FeOOH	33 h	30 min	0.67–3333.33	0.42	(53)
detection of arsenic in mineral water, spring water and tap water samples in the laboratory with nano Au-CRVfilm-modified GCE			4–40	0.2	(15)
electrochemical determination of arsenic using silver nanoparticles	11 h	5 min	0.6–2.6	0.179	(30)
electrochemical determination of arsenic(III) on mercaptoethylamine-modified Au electrode in neutral media	1 h 4 min	6 min	0.2–300	0.02	(31)
electrochemical determination of arsenic in natural waters using carbon fiber ultra-microelectrodes modified with gold nanoparticles	1 h 20 min	20 s	5–60	0.9	(24)
electrochemical sensing platform for the determination of arsenite and arsenate using electroactive nanocomposite electrodes	15 h		0.08–15	0.021–0.034	(32)
heavy metal analysis in water with a biodegradable impedimetric electrochemical sensor	sensor preparation: 5 min, drying: 6 h	40 min	1–100	1	this work

The ultimate goal in the development of this sensor methodology is to develop a sensor platform that can detect heavy metals in water and has the least harm to the environment and living things. Currently, environmentally friendly gelatin^47^/glycerol^48^ hydrogel^49^ structure is used in the main structure of the sensor. However, ME, which is used as a receptor in the sensor structure, is unfortunately not eco-friendly. Cysteine,^50^ glutathione,^51^ and dithiol peptides,^52^ which can be used instead of ME, will enable to reach the goal of eco-friendly biosensors in future studies.

## Conclusions

The presence of heavy metals in drinking water and long-term exposure to them can cause serious health problems.^54,55^ Especially for developing countries, a simple, fast, sensitive, relatively low cost, and biodegradable arsenic detection system may be useful. Real-time, fast, early, and specific detection performances in the nanogram range at LD limits with high sensitivity in water management centers and not compromising speed and cost compared to currently used detection tools and technologies under development is an important requirement.

Impedimetric electrochemical sensor is the most suitable platform for detection within 40 min without nonspecific connections. In situ, it has real-time, fast, low-cost, and high-sensitivity detection performance at LD limits in the nanogram per mL range. It is easy to design and use, especially in water management centers, enabling early, reliable, and portable transportation. This platform also has the feature of a green sensor with minimum harm to nature and the potential to dissolve in nature.

In this study, which focuses on the determination of the amount of inorganic arsenic in the range of 1–100 ng/mL using a gelatin-based solid electrochemical sensor enriched with ME and the production of the most suitable electrode for this purpose, the detection of arsenic with 1 ng/mL LOD has been proven in both DIW and drinking water. With the electrode production procedure followed in the study, a sensor platform with a wide linear range and suitable for field detection was established.

The main objective of the present study is to provide a real-time, fast, high sensitivity, and low-cost impedimetric electrochemical sensor which is employed on-site. With the international patent application filed, this novel prospective green biosensor is aimed to perform heavy metal analysis in drinking water, to monitor water quality, to perform trace metal and anion analysis in environmental samples, to determine metal concentration, to perform biosensing, and to monitor biomolecular interactions in human and animal samples, to detect biomarkers, microorganisms, DNA, proteins, and enzymes in human and animal liquid samples, to detect microorganisms and biological/chemical markers in liquid, food, and environmental samples, to perform quantitative detection of bacteria and biomarkers, and to determine DNA.

The sensor has technical advantages and gives very stable and sensitive results; Detection of arsenic with the impedimetric electrochemical prospective green biosensor with gelatin-based solid electrolyte has been performed for the first time. It provides on-site, real-time, fast, and early detection at LD limits unlike currently used methods and has no requirement of highly skilled personnel and expensive and complex instrumentation. It has high sensitivity and is low cost and easy to design and use. The system has the potential to be used as a point-of-care biosensor, allowing to perform early and reliable detection on-site, especially in water management plants, by using gelatin and glycerol chemicals for a period of approximately 40 min with a sensitivity of 1 ng/mL by performing impedance monitoring with the impedimetric electrochemical sensor having gelatin-based solid electrolyte enriched with ME and an impedance analyzer, which allows portable transportation such that nonspecific connection will not be made.

## Experimental Section

### Chemical Agents

Commercial colorless gelatin (Bovine gelatin 100%—Dr. Oetker), trademark glycerol (glycerin 80%—Health), ME (99%, Merck), ethyl alcohol (100%, Merck), potassium hydroxide (KOH—85%, Merck), PAO (97%, Sigma-Aldrich), stock solution + 99.33 (v/v) DIW, and trademark drinking water were used.

### Electrode Fabrication

In order to increase the performance of electrochemical measurement, electrode structures used in the detection and determination of important chemical compounds should be chosen based on their easy preparation method, compatibility, reliability, and wide electroanalytical applications.^56−67^

The richness of natural polymers in nature, the very low costs, and their biodegradable properties are particularly interesting. They improve the corrosion resistance and limited current density of gelatin and reduce the potential at the same current density, which seem to help.^68−72^ Hydrophilic groups in membranes composed of natural polymers such as gelatin increase the ionic conductivity. They are obtained by hydrolysis of collagen and contains high levels of glycine, proline, and hydroxyproline amino acids in its structure. They are nontoxic, biocompatible, and compatible with aqueous solvents, and due to their conductivity, they are suitable for use in electronic systems. No structure is necessary for gel formation. Mechanically, it is quite robust. It also has thermal stability, and the melting point is high enough for the gel to work. The hydroxyl groups on the gelatin were used both to bind the ME and to form plasticization with glycerol. Three hydroxyl groups in glycerol and the hydroxyl groups located at different spots on gelatin molecules undergo hydroxyl condensation reaction to form the gelatin-based electrode structure.

It is used as a cross-linking agent in biosensor applications to react the gelatin-based electrode with PAO and ME with a thiol, and a hydroxyl group was added to the electrode mixture during the electrode synthesis. The addition of ME during the electrode synthesis stage was done to provide the basic environment between pH = 8.5 and 9.0 for the hydroxyl condensation reaction without the use of extra substance for pH adjustment^73^ and to form the gelatin-based PAO reactive electrode to which PAO will be attached.

Considering the preparation of solid polymeric electrodes, 2 g of gelatin was dispersed in 15 mL of DIW at room temperature. Then, 1.75 g of plasticizer was added to this solution by mixing, and the resulting solution was poured into Petri plates to form transparent films.

Afterward, for the detection of arsenic, samples at different concentrations were prepared according to the information obtained from the literature, and the optimum sensor structure was determined. In studies carried out to determine the sensor structure, gelatin and the amount of glycerol is 1.25–1.5–1.75 and 2 g; experimental studies were carried out by determining the amount of pure water to be 10–15–20 mL.

### Electrode Characterization and Optimization

In order to determine the optimum amount of glycerol, gelatin, and DIW forming the electrode structure, experimental studies were started by considering the ratios in the literature, and gelatin-based solid electrolyte films with different ME and glycerol ratios were continued to be formed for determination.^74^ First of all, the amount of gelatin (2 g) and DIW (15 mL) were kept constant, and the glycerol amounts were changed to 1.25, 1.5, 1.75, and 2 g. In the next step, the amount of glycerol was kept constant, and the amount of gelatin was changed to 1.25, 1.5, 1.75, and 2 g. In the last step, the amount of DIW was changed to 10, 15, and 20 mL, respectively, while the amounts of gelatin (2 g) and glycerol (1.75 g) were kept constant. According to these determined amounts, nine elliptical samples of 1.5 cm × 0.5 cm dimensions were prepared, and pH measurements of all samples weredone. It was observed that the pH was in the range of 8.5–8.9. One sample of the prepared batches was left to dry for 6 h at room temperature, and one sample of each was kept at +4 °C.

Before impedance monitoring via the impedance analyzer for arsenic detection, wax was melted and poured into the bottom of the Petri dish to prevent water and arsenic from going under the sample, and only enough space was left for the sensor to enter. Liquids were applied to the middle of the sensors so that they do not affect the areas where the electrodes are located.

The impedance changes of the sample solutions transferred with a Pasteur pipette on the prepared sensor in the experiments were followed with an impedance analyzer.

To form the bioactive layer of the biosensor, gelatin was dissolved in pure water for complete dissolution. Glycerol, which was used for the gelatin structure to gain flexibility and hardness after the dissolution process, and ME, which was previously shown to be used in the literature for arsenic detection,^75^ were added to the gelatin solution for cross-linking. For ME immobilization, different concentrations (0.05, 0.1, 0.15, and 0.2) of ME (v/v) solution and 0.05 DIW (v/v) in 3.3 of ethanol (v/v) at pH = 9.0 were applied. In order to adjust the pH, 0.66 of potassium hydroxide (v/v) was added to the solution during the preparation of the ME solution. Chemical cross-linking is used to control hardness to improve the brittle mechanical property. After this step, solid electrolyte preparation is completed.

**Figure 6:**
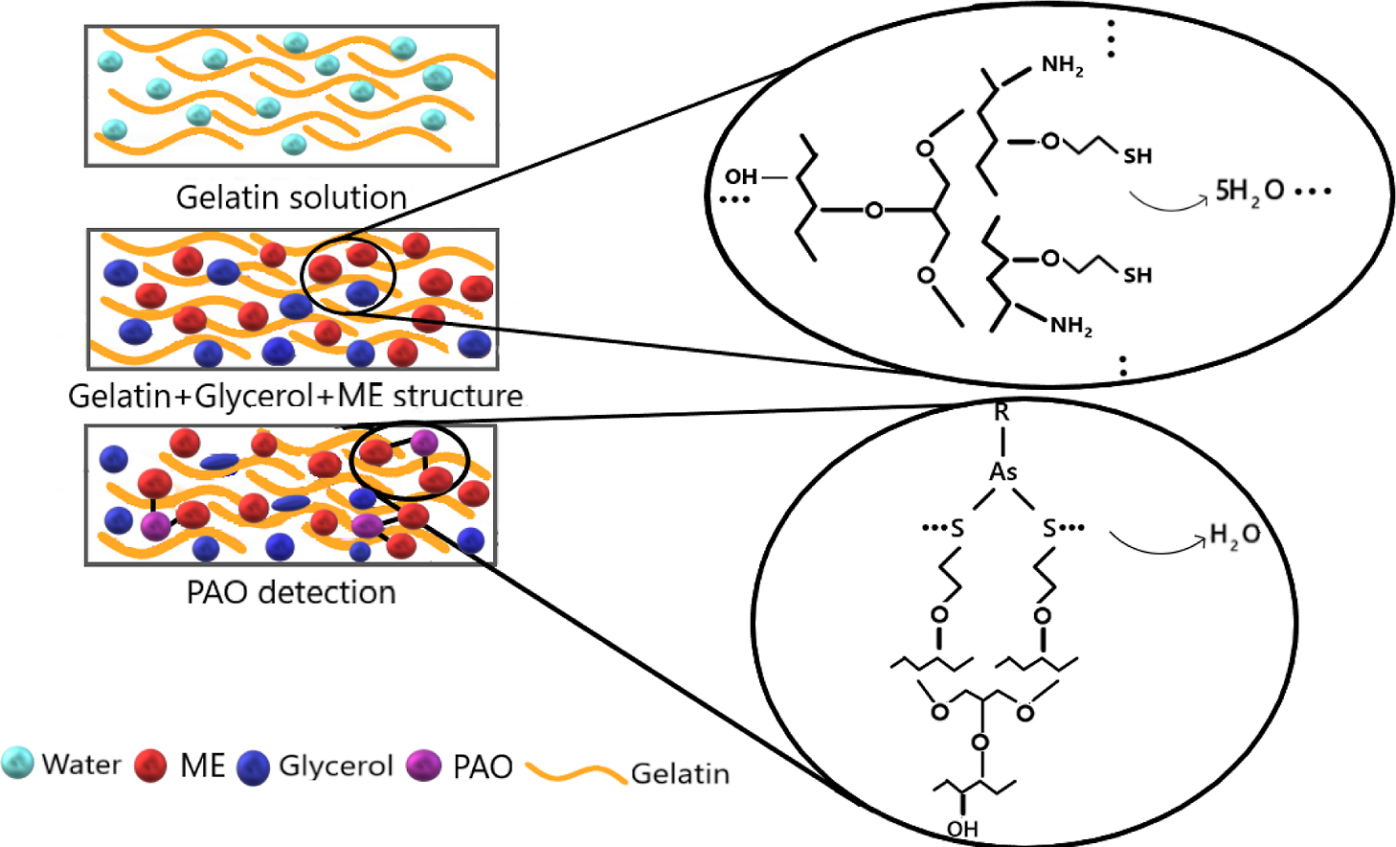
PAO detection with synthesized electrodes. After making sure that the samples were completely dry, conductivity measurements of moist and dry samples were taken.

As a result of the structure formed by the interaction of gelatin–glycerol–ME, condensation of hydroxyl groups on gelatin molecules and hydroxyl groups on free ME molecules continues, and an upper layer consisting of thiol groups forms on the surface (Figure 6).

Using an impedimetric electrochemical sensor with ME-enriched gelatin-based solid electrolyte for the detection of arsenic, it is necessary to determine the procedure that gives the most optimal result for impedance monitoring via the impedance analyzer. The effects of the amount of ME, glycerol, and gelatin and the application time on the impedimetric electrochemical sensor response were determined. In order to determine the procedure that gives the most optimal result for impedance monitoring, the optimum amount of each parameter was determined, and the impedimetric electrochemical sensor was prepared and the intended measurements were taken.

For electrode characterization and optimization, first, conductivity measurements were performed with a multimeter (Fluke-15B+) and an impedance analyzer (AIM4170C), and then impedance measurements were made using an impedance analyzer. The chemical and morphological properties of the solid electrolyte surface are directly related to the quality and sensitivity of the detection. Therefore, the morphology and surface properties of the gelatin polymer electrolyte were characterized by SEM (Zeıss-Gemini 500) and FT-IR (Bruker/Alpha).

**Figure 7:**
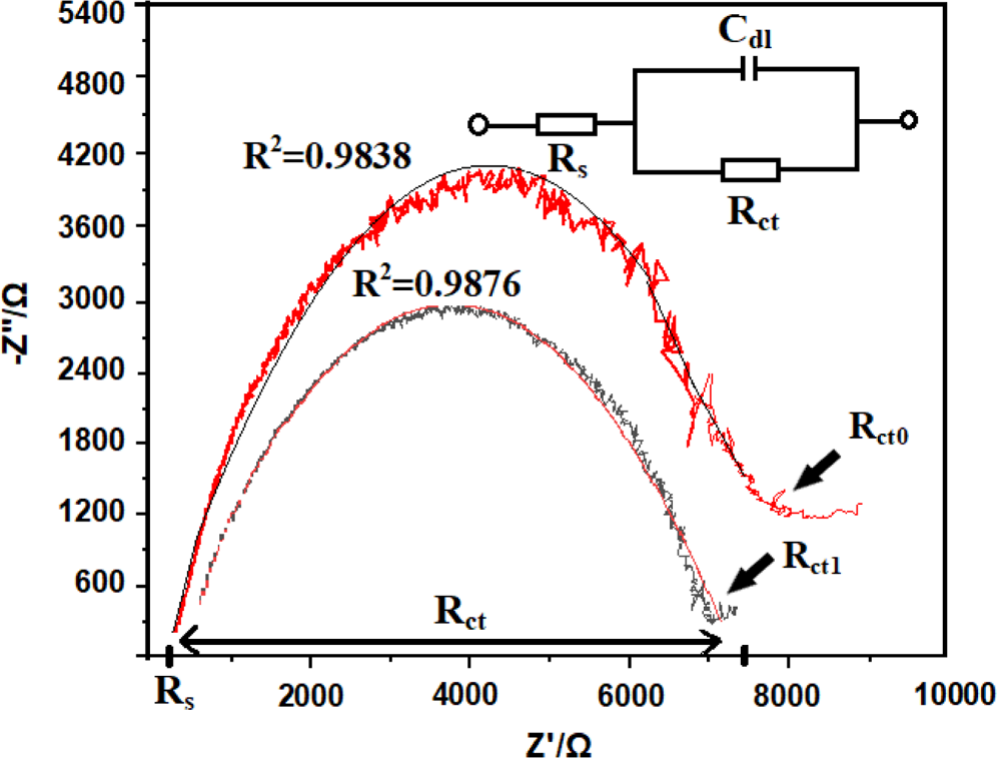
Nyquist plot and the equivalent circuit model. *Colored solid lines indicate fitted data.

It is well known that EIS is an effective tool to reflect the functionalization of the electrode step by step and monitor the feature of the surface.^76^ EIS data were first fitted, an equivalent electrical circuit model given in Figure 7 was created, and the data were examined.

In the Randles equivalent circuit structure for measurement of impedance change in the frequency range 200 kHz–10 MHz in Figure 7, *R*_s_ is the solution resistance, *R*_ct_ is the load-transfer resistance, and *C*_dl_ is the double-layer capacitance. The Nyquist plot can be explained by this equivalent circuit. Typical EIS spectrum comprises a compressed semicircle in the high-frequency region followed by a tail in the lower-frequency region. The diameter of the semicircle corresponds to the electron-transfer resistance (*R*_ct_). The high-frequency region is dominated by the double-layer capacitance (*C*) and the electrolyte solution resistance (*R*_s_).^38^

In order to determine the *R* square values, the data were fitted with the fitting algorithm in the analysis section of the Origin 2018 program. The *R* square values of the fitting were found to be 0.9838 and 0.9876 for *R*_ct0_ and *R*_ct1_ curves, respectively.

Percentage resistance change [Δ*R*_ct_ (%)] values in the presentation of numerical expressions in the article were calculated according to eq 1. *R*_ct0_ in eq 1 shows the initial *R*_ct_ when DIW is added to the prepared sensor, while the *R*_ct1_ value shows the latest *R*_ct_ after detection.
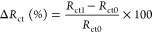
1

### PAO Detection

In the arsenic detection study, PAO, which is the most common type of arsenic pollution in groundwater, was preferred.^77^ For the determination of PAO, the medium was determined as DIW, and first, a stock solution was prepared by adding 1 mg of PAO to 50 mL of DIW. Then, necessary dilutions were made from this solution, and 100, 10 and 1 ng/mL PAO solutions were obtained, respectively. 0.5 mL of PAO solutions prepared in drinking water and DIW were taken and applied to the sensor surface for 40 min, and the measurements were quantified by monitoring the Δ*R*_ct_ change. Each experiment was repeated at least three times for three different concentrations to obtain the means and standard deviations of the Δ*R*_ct_ change. The impedance change was monitored with an impedance analyzer using the built-in signal processing algorithm, which provides reliable detection of the impedance change in the frequency range 200 kHz–10 MHz.

## References

[ref1] Mandal B. K.; Suzuki K. T. Arsenic Round the World: A Review. Talanta 2002, 58, 201–235. 10.1016/S0039-9140(02)00268-0. 18968746

[ref2] Liu Z. G.; Huang X. J. Voltammetric Determination of Inorganic Arsenic. TrAC, Trends Anal. Chem. 2014, 60, 25–35. 10.1016/J.TRAC.2014.04.014.

[ref3] Kumaresan M.; Riyazuddin P. Overview of Speciation Chemistry of Arsenic. Curr. Sci. 2001, 80, 837–846.

[ref4] Salek Maghsoudi A. S.; Hassani S.; Mirnia K.; Abdollahi M. Recent Advances in Nanotechnology-Based Biosensors Development for Detection of Arsenic, Lead, Mercury, and Cadmium. Int. J. Nanomed. 2021, 16, 803–832. 10.2147/IJN.S294417. PMC7870343 33568907

[ref5] Rodríguez-Lado L.; Sun G.; Berg M.; Zhang Q.; Xue H.; Zheng Q.; Johnson C. A. Groundwater Arsenic Contamination throughout China. Science 2013, 341, 866–868. 10.1126/science.1237484. 23970694

[ref6] Mehdi S. E. H.; Amen R.; Ali A.; Anjum H.; Mahmood A.; Mubashir M.; Mukhtar A.; Ullah S.; Al-Sehemi A. G.; Ibrahim M.; Khan M. S.; Qyyum M. A.; Show P. L. Sources, Chemistry, Bioremediation and Social Aspects of Arsenic-Contaminated Waters: A Review. Environ. Chem. Lett. 2021, 19, 3859–3886. 10.1007/s10311-021-01254-3.

[ref7] Ning Z.; Lobdell D. T.; Kwok R. K.; Liu Z.; Zhang S.; Ma C.; Riediker M.; Mumford J. L. Residential Exposure to Drinking Water Arsenic in Inner Mongolia, China. Toxicol. Appl. Pharmacol. 2007, 222, 351–356. 10.1016/j.taap.2007.02.012. 17448512

[ref8] Feeney R.; Kounaves S. P. On-Site Analysis of Arsenic in Groundwater Using a Microfabricated Gold Ultramicroelectrode Array. Anal. Chem. 2000, 72, 2222–2228. 10.1021/ac991185z. 10845367

[ref9] Tchounwou P. B.; Patlolla A. K.; Centeno J. A. Invited Reviews: CarcinogeniC and Systemic Health Effects Associated with Arsenic Exposure—A Critical Review. Toxicol. Pathol. 2003, 31, 575–588. 10.1080/01926230390242007. 14585726

[ref10] Shi H.; Shi X.; Liu K. J.; Liu K. J. Oxidative Mechanism of Arsenic Toxicity and Carcinogenesis. Mol. Cell. Biochem. 2004, 255, 67–78. 10.1023/b:mcbi.0000007262.26044.e8. 14971647

[ref11] Melamed D. Monitoring Arsenic in the Environment: A Review of Science and Technologies with the Potential for Field Measurements. Anal. Chim. Acta 2005, 532, 1–13. 10.1016/j.aca.2004.10.047.

[ref12] Rahman M. R.; Okajima T.; Ohsaka T. Selective Detection of As(III) at the Au(111)-like Polycrystalline Gold Electrode. Anal. Chem. 2010, 82, 9169–9176. 10.1021/ac101206j. 20973517

[ref13] Mays D. E.; Hussam A. Voltammetric Methods for Determination and Speciation of Inorganic Arsenic in the Environment-A Review. Anal. Chim. Acta 2009, 646, 6–16. 10.1016/j.aca.2009.05.006. 19523550

[ref14] Sengupta M. K.; Sawalha M. F.; Ohira S. I.; Idowu A. D.; Dasgupta P. K. Green Analyzer for the Measurement of Total Arsenic in Drinking Water: Electrochemical Reduction of Arsenate to Arsine and Gas Phase Chemiluminescence with Ozone. Anal. Chem. 2010, 82, 3467–3473. 10.1021/ac100604y. 20380446

[ref15] Rajkumar M.; Thiagarajan S.; Chen S.-M. Electrochemical Detection of Arsenic in Various Water Samples. Int. J. Electrochem. Sci. 2011, 6, 3164–3177.

[ref16] Matschullat J.; Perobelli Borba R.; Deschamps E.; Figueiredo B. R.; Gabrio T.; Schwenk M. Human and Environmental Contamination in the Iron Quadrangle, Brazil. Appl. Geochem. 2000, 15, 181–190. 10.1016/s0883-2927(99)00039-6.

[ref17] Reddy R. R.; Rodriguez G. D.; Webster T. M.; Abedin M. J.; Karim M. R.; Raskin L.; Hayes K. F. Evaluation of Arsenic Field Test Kits for Drinking Water: Recommendations for Improvement and Implications for Arsenic Affected Regions Such as Bangladesh. Water Res. 2020, 170, 115325 10.1016/j.watres.2019.115325. 31785563

[ref18] Zounr R. A.; Tuzen M.; Khuhawar M. Y. Ultrasound Assisted Deep Eutectic Solvent Based on Dispersive Liquid Liquid Microextraction of Arsenic Speciation in Water and Environmental Samples by Electrothermal Atomic Absorption Spectrometry. J. Mol. Liq. 2017, 242, 441–446. 10.1016/j.molliq.2017.07.053.

[ref19] Acharya R.; Nair A. G. C.; Reddy A. V. R. Speciation and Instrumental Neutron Activation Analysis for Arsenic in Water Samples. J. Radioanal. Nucl. Chem. 2009, 281, 279–282. 10.1007/s10967-009-0101-z.

[ref20] B’Hymer C.; Caruso J. A. Arsenic and Its Speciation Analysis Using High-Performance Liquid Chromatography and Inductively Coupled Plasma Mass Spectrometry. J. Chromatogr. 2004, 1045, 1–13. 10.1016/j.chroma.2004.06.016. 15378873

[ref21] Majid E.; Hrapovic S.; Liu Y.; Male K. B.; Luong J. H. T. Electrochemical Determination of Arsenite Using a Gold Nanoparticle Modified Glassy Carbon Electrode and Flow Analysis. Anal. Chem. 2006, 78, 762–769. 10.1021/ac0513562. 16448049

[ref22] Luvonga C.; Rimmer C. A.; Yu L. L.; Lee S. B. Determination of Total Arsenic and Hydrophilic Arsenic Species in Seafood. J. Food Compos. Anal. 2021, 96, 103729 10.1016/j.jfca.2020.103729. PMC8174234 34092915

[ref23] Kempahanumakkagari S.; Deep A.; Kim K. H.; Kumar Kailasa S.; Yoon H. O. Nanomaterial-Based Electrochemical Sensors for Arsenic - A Review. Biosens. Bioelectron. 2017, 95, 106–116. 10.1016/j.bios.2017.04.013. 28431363

[ref24] Carrera P.; Espinoza-Montero P. J.; Fernández L.; Romero H.; Alvarado J. Electrochemical Determination of Arsenic in Natural Waters Using Carbon Fiber Ultra-Microelectrodes Modified with Gold Nanoparticles. Talanta 2017, 166, 198–206. 10.1016/j.talanta.2017.01.056. 28213223

[ref25] Devi P.; Thakur A.; Lai R. Y.; Saini S.; Jain R.; Kumar P. Progress in the Materials for Optical Detection of Arsenic in Water. TrAC, Trends Anal. Chem. 2019, 110, 97–115. 10.1016/j.trac.2018.10.008.

[ref26] Sadrolhosseini A. R.; Naseri M.; Kamari H. M. Surface Plasmon Resonance Sensor for Detecting of Arsenic in Aqueous Solution Using Polypyrrole-Chitosan-Cobalt Ferrite Nanoparticles Composite Layer. Opt. Commun. 2017, 383, 132–137. 10.1016/j.optcom.2016.08.065.

[ref27] Al-Rekabi S. H.; Mustapha Kamil Y.; Abu Bakar M. H.; Fen W. F.; Lim H. N.; Kanagesan S.; Mahdi M. A. Hydrous Ferric Oxide-Magnetite-Reduced Graphene Oxide Nanocomposite for Optical Detection of Arsenic Using Surface Plasmon Resonance. Opt. Laser Technol. 2019, 111, 417–423. 10.1016/j.optlastec.2018.10.018.

[ref28] Xu C.; Liu D.; Zhang D.; Zhao C.; Liu H. Ultrasensitive Point-of-Care Testing of Arsenic Based on a Catalytic Reaction of Unmodified Gold Nanoparticles. New J. Chem. 2018, 42, 14857–14862. 10.1039/c8nj03259a.

[ref29] U P.; KM A. G.; MG E.; B S. T.; N N.; B R. M. Biologically Synthesized PbS Nanoparticles for the Detection of Arsenic in Water. Int. Biodeterior. Biodegrad. 2017, 119, 78–86. 10.1016/j.ibiod.2016.10.009.

[ref30] Sonkoue B. M.; Tchekwagep P. M. S.; Nanseu-Njiki C. P.; Ngameni E. Electrochemical Determination of Arsenic Using Silver Nanoparticles. Electroanalysis 2018, 30, 2738–2743. 10.1002/elan.201800520.

[ref31] Li D.; Li J.; Jia X.; Han Y.; Wang E. Electrochemical Determination of Arsenic(III) on Mercaptoethylamine Modified Au Electrode in Neutral Media. Anal. Chim. Acta 2012, 733, 23–27. 10.1016/j.aca.2012.04.030. 22704371

[ref32] Gumpu M. B.; Veerapandian M.; Krishnan U. M.; Rayappan J. B. B. Electrochemical Sensing Platform for the Determination of Arsenite and Arsenate Using Electroactive Nanocomposite Electrode. Chem. Eng. J. 2018, 351, 319–327. 10.1016/j.cej.2018.06.097.

[ref33] Kalambate P. K.; Rao Z.; Dhanjai; Wu J.; Shen Y.; Boddula R.; Huang Y. Electrochemical (Bio) Sensors Go Green. Biosens. Bioelectron. 2020, 163, 112270 10.1016/j.bios.2020.112270. 32568692

[ref34] Hoare D.; Bussooa A.; Neale S.; Mirzai N.; Mercer J. The Future of Cardiovascular Stents: Bioresorbable and Integrated Biosensor Technology. Adv. Sci. 2019, 6, 1900856 10.1002/advs.201900856. PMC6794628 31637160

[ref35] Boutry C. M.; Schroeder B. C.; Bao Z.; Legrand A.; Fox P. A Sensor Measuring Deformation and Pressure, Entirely Biodegradable, for Orthopedic Applications. Proceedings of the 2016 IEEE Biomedical Circuits and Systems Conference (BioCAS), 2016; pp 144–147.

[ref36] De Santis M.; Cacciotti I. Wireless Implantable and Biodegradable Sensors for Postsurgery Monitoring: Current Status and Future Perspectives. Nanotechnology 2020, 31, 252001 10.1088/1361-6528/ab7a2d. 32101794

[ref37] Park S.-M.; Yoo J.-S. Peer Reviewed: Electrochemical Impedance Spectroscopy for Better Electrochemical Measurements. Anal. Chem. 2003, 75, 455–461. 10.1021/ac0313973. 14619851

[ref38] Lisdat F.; Schäfer D. The Use of Electrochemical Impedance Spectroscopy for Biosensing. Anal. Bioanal. Chem. 2008, 391, 1555–1567. 10.1007/s00216-008-1970-7. 18414837

[ref39] Stanković A.; Kajinić Ž.; Turkalj J. V.; Romić Ž.; Sikirić M. D.; Asserghine A.; Nagy G.; Medvidović-Kosanović M. Voltammetric Determination of Arsenic with Modified Glassy Carbon Electrode. Electroanalysis 2020, 32, 1043–1051. 10.1002/elan.201900666.

[ref40] Zhang X.; Wang S. Voltametric Behavior of Noradrenaline at 2-Mercaptoethanol Self-Assembled Monolayer Modified Gold Electrode and Its Analytical Application. Sensors 2003, 3, 61–68. 10.3390/s30300061.

[ref41] Mushiana T.; Mabuba N.; Idris A. O.; Peleyeju G. M.; Orimolade B. O.; Nkosi D.; Ajayi R. F.; Arotiba O. A. An Aptasensor for Arsenic on a Carbon-gold Bi-Nanoparticle Platform. Sens. Bio-Sens. Res. 2019, 24, 100280 10.1016/j.sbsr.2019.100280.

[ref42] Liu C.; Zhang H. J.; You X.; Cui K.; Wang X. Electrically Conductive Tough Gelatin Hydrogel. Adv. Electron. Mater. 2020, 6, 2000040 10.1002/aelm.202000040.

[ref43] Ghadami A.; Taheri Qazvini N.; Nikfarjam N. Ionic Conductivity in Gelatin-Based Hybrid Solid Electrolytes: The Non-Trivial Role of Nanoclay. J. Mater. Sci. Technol. 2014, 30, 1096–1102. 10.1016/j.jmst.2014.06.008.

[ref44] Joseph L.; Jun B. M.; Flora J. R. V.; Park C. M.; Yoon Y. Removal of Heavy Metals from Water Sources in the Developing World Using Low-Cost Materials: A Review. Chemosphere 2019, 229, 142–159. 10.1016/j.chemosphere.2019.04.198. 31078029

[ref45] Corsello S.; Fulgenzi A.; Vietti D.; Ferrero M. E. The Usefulness of Chelation Therapy for the Remission of Symptoms Caused by Previous Treatment with Mercury-Containing Pharmaceuticals: A Case Report. Cases J. 2009, 2, 199 10.1186/1757-1626-2-199. 19946446 PMC2783151

[ref46] Vilensky J. A.; Redman K. British Anti-Lewisite (Dimercaprol): An Amazing History. Ann. Emerg. Med. 2003, 41, 378–383. 10.1067/mem.2003.72. 12605205

[ref47] Li X.; Jiang G.; Yang L.; Wang K.; Shi H.; Li G.; Wu X. Application of Gelatin Quaternary Ammonium Salt as an Environmentally Friendly Shale Inhibitor for Water-Based Drilling Fluids. Energy Fuels 2019, 33, 9342–9350. 10.1021/acs.energyfuels.9b01798.

[ref48] Chen Y.; Ma Y.; Lu W.; Guo Y.; Zhu Y.; Lu H.; Song Y. Environmentally Friendly Gelatin/β-Cyclodextrin Composite Fiber Adsorbents for the Efficient Removal of Dyes from Wastewater. Molecules 2018, 23, 2473 10.3390/molecules23102473. 30261678 PMC6222675

[ref49] Athanasiadis V.; Grigorakis S.; Lalas S.; Makris D. P. Highly Efficient Extraction of Antioxidant Polyphenols from Olea Europaea Leaves Using an Eco-Friendly Glycerol/Glycine Deep Eutectic Solvent. Waste Biomass Valorization 2018, 9, 1985–1992. 10.1007/s12649-017-9997-7.

[ref50] Qiu P.; Yang H.; Song Y.; Yang L.; Lv L.; Zhao X.; Ge L.; Chen C. Potent and Environmental-Friendly L-Cysteine @ Fe2O3 Nanostructure for Photoelectrochemical Water Splitting. Electrochim. Acta 2018, 259, 86–93. 10.1016/j.electacta.2017.10.168.

[ref51] Pham T. A.; Kim J. S.; Kim J. S.; Jeong Y. T. One-Step Reduction of Graphene Oxide with l-Glutathione. Colloids Surf., A 2011, 384, 543–548. 10.1016/j.colsurfa.2011.05.019.

[ref52] Chen S.; Gopalakrishnan R.; Schaer T.; Marger F.; Hovius R.; Bertrand D.; Pojer F.; Heinis C. Dithiol Amino Acids Can Structurally Shape and Enhance the Ligand-Binding Properties of Polypeptides. Nat. Chem. 2014, 6, 1009–1016. 10.1038/nchem.2043. 25343607

[ref53] Wang L.; Xu X.; Niu X.; Pan J. Colorimetric Detection and Membrane Removal of Arsenate by a Multifunctional L-Arginine Modified FeOOH. Sep. Purif. Technol. 2021, 258, 118021 10.1016/j.seppur.2020.118021.

[ref54] Wu X.; Cobbina S. J.; Mao G.; Xu H.; Zhang Z.; Yang L. A Review of Toxicity and Mechanisms of Individual and Mixtures of Heavy Metals in the Environment. Environ. Sci. Pollut. Res. 2016, 23, 8244–8259. 10.1007/s11356-016-6333-x. 26965280

[ref55] Tchounwou P. B.; Yedjou C. G.; Patlolla A. K.; Sutton D. J. Heavy Metal Toxicity and the Environment. Exper. Suppl. 2012, 101, 133–164. 10.1007/978-3-7643-8340-4_6. 22945569 PMC4144270

[ref56] Mazloum-Ardakani M.; Taleat Z. Investigation of Electrochemistry Behavior of Hydroxylamine at Glassy Carbon Electrode by Indigocarmine. Int. J. Electrochem. Sci. 2009, 4, 694–706.

[ref57] Thiagarajan S.; Chen S.-M.; Lin K.-H. Electrochemical Preparation of VPtCl[Sub 6] Film and Its Electrocatalytic Properties with NAD[Sup +] and Sulfur Oxoanions. J. Electrochem. Soc. 2008, 155, E33–E41. 10.1149/1.2830846.

[ref58] Su B. W.; Thiagarajan S.; Chen S. M. The Interaction of Iodide Film with Platinum Microparticles on Different Electrode Materials for Various Electrocatalytic Reactions. Electroanalysis 2008, 20, 1987–1995. 10.1002/elan.200804275.

[ref59] Zare H. R.; Zare H. R.; Samimi R.; Ardakani M. M. A Comparison of the Electrochemical Behavior of Rutin at an Inactivated, Activated, and Multi Wall Carbon Nanotubes Modified Glassy Carbon Electrode. Int. J. Electrochem. Sci. 2009, 4, 730–739.

[ref60] Zhao F.; Guo G.; Xiao F.; Zeng B. Voltammetric Determination of Tetracycline by Using Multi-Wall Carbon Nanotube-Ionic Liquid Film Coated Glassy Carbon Electrode. Int. J. Electrochem. Sci. 2009, 4, 1365–1372.

[ref61] Tsai T. H.; Thiagarajan S.; Chen S. M. Green Synthesized Au-Ag Bimetallic Nanoparticles Modified Electrodes for the Amperometric Detection of Hydrogen Peroxide. J. Appl. Electrochem. 2010, 40, 2071–2076. 10.1007/s10800-010-0188-5.

[ref62] Cheng C.-Y.; Thiagarajan S.; Chen S.-M. Electrochemical Fabrication of AuRh Nanoparticles and Their Electroanalytical Applications. Int. J. Electrochem. Sci. 2011, 6, 1331–1341.

[ref63] Rajkumar M.; Thiagarajan S.; Chen S.-M. Electrochemical Fabrication of Rh-Pd Particles and Electrocatalytic Applications. J. Appl. Electrochem. 2011, 41, 663–668. 10.1007/s10800-011-0277-0.

[ref64] Tsai T.-H.; Wang S.-H.; Chen S.-M. Anthraquinonedisulfonate Doped Glutaraldehyde Cross-Linked Poly-L-Lysine Modified Electrode for S2O8 2-, IO3-and Oxygen Reduction. Int. J. Electrochem. Sci. 2011, 6, 1655–1668.

[ref65] Thiagarajan S.; Cheng C. Y.; Chen S.-M.; Tsai T. H. Electrochemical Detection of Propofol at the Preanodized Carbon Electrode. J. Solid State Electrochem. 2011, 15, 781–786. 10.1007/s10008-010-1160-3.

[ref66] Thiagarajan S.; Tsai T. H.; Chen S.-M. Electrochemical Fabrication of Nano Manganese Oxide Modified Electrode for the Detection of H 2 O 2. Int. J. Electrochem. Sci. 2011, 6, 2235–2245.

[ref67] Vieira D. F.; Avellaneda C. O.; Pawlicka A. Conductivity Study of a Gelatin-Based Polymer Electrolyte. Electrochim. Acta 2007, 53, 1404–1408. 10.1016/j.electacta.2007.04.034.

[ref68] Li Q.; Wang G.; Fan J.; Xu S.; Zhang J.; Chen J.; Wang R. Effect of Gelatin on Electrodeposition of Tellurium from Alkaline Electrolyte. Mater. Res. Express 2019, 6, ab4849 10.1088/2053-1591/ab4849.

[ref69] Slade L.; Levine H. Polymer-Chemical Properties of Gelatin in Foods. Adv. Meat Res. 1987, 4, 251–266.

[ref70] Bergo P.; Sobral P. J. A. Effects of Plasticizer on Physical Properties of Pigskin Gelatin Films. Food Hydrocolloids 2007, 21, 1285–1289. 10.1016/j.foodhyd.2006.09.014.

[ref71] Vieira D. F.; Pawlicka A. Optimization of Performances of Gelatin/LiBF4-Based Polymer Electrolytes by Plasticizing Effects. Electrochim. Acta 2010, 55, 1489–1494. 10.1016/j.electacta.2009.04.039.

[ref72] Soylu M. C.; Shih W. H.; Shih W. Y. Insulation by Solution 3-Mercaptopropyltrimethoxysilane (MPS) Coating: Effect of PH, Water, and Mps Content. Ind. Eng. Chem. Res. 2013, 52, 2590–2597. 10.1021/ie302231g.

[ref73] Orazem M. E.; Trıbollet B. Electrochemıcal Impedance Spectroscopy; John Wiley & Sons, 2008.

[ref74] Díaz P.; Arratia C.; Vásquez C.; Osorio F.; Enrione J. Effect of Glycerol on Water Sorption of Bovine Gelatin Films in the Glassy State. Procedia Food Sci. 2011, 1, 267–274. 10.1016/j.profoo.2011.09.042.

[ref75] Keser K.; Mıhçıokur H.; Çağrı Soylu M. Simple, Rapid and Sensitive Detection of Phenylarsine Oxide in Drinking Water Using Quartz Crystal Microbalance: A Novel Surface Functionalization Technique. ChemistrySelect 2020, 5, 2057–2062. 10.1002/slct.201904821.

[ref76] Grossi M.; Riccò B. Electrical Impedance Spectroscopy (EIS) for Biological Analysis and Food Characterization: A Review. J. Sens. Sens. Syst. 2017, 6, 303–325. 10.5194/jsss-6-303-2017.

[ref77] Daus B.; Mattusch J.; Wennrich R.; Weiss H. Analytical Investigations of Phenyl Arsenicals in Groundwater. Talanta 2008, 75, 376–379. 10.1016/j.talanta.2007.11.024. 18371894

